# Endothelial Dysfunction Correlates with Liver Fibrosis in Chronic HCV Infection

**DOI:** 10.1155/2015/682174

**Published:** 2015-04-27

**Authors:** Michele Barone, Maria Teresa Viggiani, Annabianca Amoruso, Serafina Schiraldi, Annapaola Zito, Fiorella Devito, Francesca Cortese, Michele Gesualdo, Natale Brunetti, Alfredo Di Leo, Pietro Scicchitano, Marco Matteo Ciccone

**Affiliations:** ^1^Gastroenterology Unit, Department of Medical and Surgical Science, University of Foggia, OO. RR. Foggia, Viale Pinto 1, 71122 Foggia, Italy; ^2^Gastroenterology Unit, Department of Emergency and Organ Transplantation, Ospedale Policlinico, University of Bari, Piazza G. Cesare 11, 70124 Bari, Italy; ^3^Cardiology Unit, Department of Emergency and Organ Transplantation, Ospedale Policlinico, University of Bari, Piazza G. Cesare 11, 70124 Bari, Italy; ^4^Cardiology Unit, Department of Medical and Surgical Science, University of Foggia, OO. RR. Foggia, Viale Pinto 1, 71122 Foggia, Italy

## Abstract

*Background*. Hepatitis C virus (HCV) infection can exert proatherogenic activities due to its direct action on vessel walls and/or via the chronic inflammatory process involving the liver.* Aims*. To clarify the role of HCV in atherosclerosis development in monoinfected HCV patients at different degrees of liver fibrosis and with no risk factors for coronary artery disease.* Methods*. Forty-five patients were included. Clinical, serological, and anthropometric parameters, liver fibrosis (transient liver elastometry (fibroscan) and aspartate aminotransferase to platelet ratio index (APRI)), carotid intima-media thickness (c-IMT), and brachial artery flow-mediated vasodilatation (FMD) were assessed. Patients were divided into 3 tertiles according to fibroscan values.* Results*. Patients in the third tertile (fibroscan value >11.5 KPa) showed FMD values were significantly lower than second and first tertiles (4.7 ± 1.7% versus 7.1 ± 2.8%, *p* = 0.03). FMD values were inversely related to liver elastomeric values. c-IMT values were normal. The risk for endothelial dysfunction development in the third tertile (*p* = 0.02) was 6.9 higher than the first tertile. A fibroscan value >11.5 KPa had a positive predictive power equal to 79% for endothelial dysfunction.* Conclusions*. HCV advanced liver fibrosis promotes atherosclerosis by inducing endothelial dysfunction independently of common cardiovascular risk factors.

## 1. Introduction

Despite significant advances in the treatment of coronary artery disease (CAD), this condition remains the leading cause of death worldwide [1]. In addition to conventional cardiovascular risk factors (age, sex, dyslipidemia, diabetes, hypertension, obesity, smoking, and physical inactivity), elevated C-reactive protein levels and homocysteine levels, alterations in coagulation and fibrinolysis regulatory factors, metabolic syndrome, and systemic inflammatory conditions are now considered new cardiovascular risk factors [2–4].

The role of systemic inflammation in the genesis of atherosclerosis is supported by studies on chronic infections [5] and chronic inflammatory autoimmune diseases (Hashimoto's thyroiditis, rheumatoid arthritis, systemic lupus erythematosus, psoriatic arthritis, ankylosing spondylitis, and inflammatory bowel diseases) [6, 7].

Concerning chronic liver diseases, it is already known that the nonalcoholic fatty liver disease correlates with CAD [8]. Nevertheless, conflicting data are on the relationship between CAD and hepatitis C virus- (HCV-) related chronic hepatitis. A cross-sectional study and three case-control researches found an increased risk for atherosclerosis (assessed by means of carotid intima-media thickness (C-IMT)) in HCV patients [9–12]. Therefore, HCV positiveness could be considered as a risk factor for CAD [13, 14], although conflicting data are in literature about such a matter [15, 16].

The endothelial dysfunction is the earliest event in the atherosclerotic process and it occurs when it is not yet a demonstrable morphological lesion of the vessel wall [17]. Its identification is a useful tool in early stratification of patients at risk for cardiovascular events [18–28]. The endothelial dysfunction consists of a reduced endothelium-dependent vasodilator response to ischemia and a modified procoagulant and proinflammatory endothelium activity. These alterations are attributed to an excess in oxidative stress and a reduced bioavailability of nitric oxide (NO), that is, the most important mediator of endothelial function [6]. The assessment of endothelial function can be performed using a noninvasive technique: the brachial artery “flow-mediated vasodilation” (FMD) [29].

The importance in detection of early signs of endothelial dysfunction is related to the reversibility of such a pathological condition: appropriate therapies (ACE inhibitors, angiotensin receptor blockers, statins, antioxidants, and even continuous positive airway pressure) are able to restore the protective role of the vascular endothelium, thus consequentially reversing the cardiovascular risk profile of patients [30].

At the best of our knowledge, only two studies evaluated endothelial dysfunction in HCV-related chronic hepatitis, although the enrolled patients were HIV coinfected [31, 32].

Our study is the first that tried to noninvasively evaluate (by means of FMD and C-IMT) early impairment in cardiovascular risk profile in HCV monoinfected patients at different stages of liver fibrosis, assessed by transient liver elastography (fibroscan) and aspartate aminotransferase to platelet ratio index (APRI) (two parameters widely accepted as indirect indexes of liver fibrosis)  [33, 34]. Strict selection criteria were used in order to enrol HCV patients without any other cardiovascular risk factor.

## 2. Material and Methods

### 2.1. Study Design

This study was performed between April 2012 and April 2013 in the Gastroenterology Unit and in collaboration with the Angiology Section of the Cardiology Unit, Ospedale Policlinico, University of Bari, Italy. A total of 380 consecutive subjects with HCV-related chronic hepatitis and followed as outpatients were considered.

Inclusion criteria were as follows: HCV monoinfection, no antiviral treatment during the last 12 months, and age between 40 and 75 years. Exclusion criteria were as follows: HBV or HIV coinfection, alcohol abuse, age <40 or >75 years, antiviral treatment in progress or suspended from less than 1 year, hepatocellular carcinoma and liver failure, coexistence of other chronic inflammatory diseases, autoimmune disease, and the presence of cardiovascular risks. Cardiovascular risk factors (documented cardiovascular disease, previous myocardial infarction or stroke, peripheral arterial disease, history of coronary revascularization procedures, moderate-to-severe chronic renal failure, hypertension, dyslipidemia, and diabetes mellitus) were defined according to European Society of Cardiology guidelines [35]. The European Low Risk Chart (SCORE) was used in order to identify patients with a probability >5% of experiencing a fatal cardiovascular event over 10 years [36].

In relation to our strict selection criteria, only 45 patients suffering from HCV-related chronic hepatitis at various stages of fibrosis and with a low cardiovascular risk were finally enrolled.

In particular, the final number of patients was obtained as follows: 7 patients refused consent for being included in this study; 4 refused to undergo FMD evaluation; 27 patients were <40 or >75 years old; 20 patients suffered from coronary heart diseases (defined as previous coronary angiography showing significant coronary artery stenosis, i.e., stenosis >50%, revascularization therapy by means of percutaneous intervention, and/or coronary artery bypass surgery); 9 patients suffered from autoimmune diseases. Furthermore, when considering the liver diseases, we excluded the following number of patients: 35 were excluded due to their alcohol addiction; 48 patients suffered from HBV/HCV coinfection; 62 showed hepatocellular carcinoma in form of one or multiple nodules in the liver. Finally, 123 patients underwent antiviral treatment within the last year before enrolment. Therefore, from the original number of 380 patients, the final number of enrolled patients was 45.

Informed consent was obtained from each patient before enrolment. The study was performed in agreement with the ethical guidelines of the Declaration of Helsinki and received the approval by the local Ethic Committee.

Each patient was evaluated according to age, blood pressure, body mass index (BMI), waist circumference, and ongoing therapy. In addition, transaminase, total cholesterol, platelets, quantitative HCV-RNA, and HCV-RNA genotype were assessed.  Table 1 gathers all the main characteristics of the population of the study.

### 2.2. Liver Stiffness Evaluation

The liver stiffness was assessed at the enrolment phase by the evaluation of transient liver elastometry (liver stiffness), using a fibroscan 502 apparatus (Echosens, Paris, France), and APRI.

Fibroscan was performed as reported by Sandrin et al. [33], using at least 10 valid measurements. Examinations were considered reliable only when interquartile range (IQR) was <30% and the success rate (SR) was >60%.

We referred to Tsochatzis et al. to stage liver fibrosis by fibroscan [37]. They identified the following range in HCV-related chronic hepatitis: F0-F1 (<7.6 KPa), F2 (between 7.6 and 10.9 KPa), F3 (between 10.9 and 15.3 KPa), and F4 (>15.3 KPa).

According to the fibroscan value, patients were divided into three tertiles. The first one consisted of 15 patients with a fibroscan value ranging from 3.5 to 6.2 KPa (mean 4.9 ± 0.8 KPa), the second tertile was composed by 15 patients with a fibroscan from 6.6 to 10.9 (mean 8.6 ± 1.4 KPa), and the third one consisted of 15 patients with a fibroscan from 11.5 to 47.6 KPa (mean 27.2 ± 12.1 KPa).

### 2.3. Evaluation of Flow-Mediated Dilation (FMD)

Temperature, food, stress, drugs, and sympathetic stimuli influence the FMD. The study was performed with the subjects fasting for at least 8–12 hours, in a quiet air conditioned room (22–24°C), early in the morning. The subjects were asked not to play exercise or take exciting substances like coffee/tea, chocolate which could impair endothelial function and for at least 4–6 hours before the exam. A preliminary scan explored the anatomy and identified landmarks, paying attention to poor quality images, the presence of atherosclerotic plaques, calcifications, arterial tortuosity, or kinking. The right brachial artery was evaluated in a long axis projection between 5 and 10 cm above the elbow using a 7.0 MHz or higher linear probe. The study was performed using a high resolution ultrasonography (Philips Sonos 5500) connected to an image analysis system, certified by the CNR of Pisa (MVE II) [38], for computing the brachial artery diameter in real time by analyzing B-mode ultrasound images, setting positivity to the test value at less than 5%. All the ultrasound examinations were performed by the same physician in order to reduce bias. With the subject in supine position for at least 10 minutes, the arm was positioned comfortably in order to get good images of the brachial artery. The selected artery segment to be displayed was above the antecubital fossa in the longitudinal plane, identifying the part where the anterior and posterior intimal interfaces between the lumen and vessel wall were clear. A sphygmomanometer cuff was placed in the distal site to the artery, in cases of a humeral artery on the forearm. After 1 minute of flow image baseline acquisition, the artery was occluded by inflating the cuff to a pressure of 200–220 mmHg for exactly 5 min. When the cuff is deflated, it induces a short state of high flow (reactive hyperemia in the forearm microcirculation) through the brachial artery to adjust to the reduced downstream resistance caused by the ischemia-induced dilatation. The resulting increased shear stress provides the stimulus for dilatation of the humeral artery. Within 15 seconds from the end of ischemia, the flow rate was measured and then the degree of hyperemia. The image of the artery was then recorded continuously for 2-3 minutes after ischemia. Reactive hyperemia was calculated as the ratio of the change in diameter (maximal dilatation after deflation baseline) divided by the baseline value, which corresponds to the maximum FMD recovery value. FMD was analyzed as the percentage increase in brachial artery diameter after the application of a pressure stimulus [39].

In order to reduce bias, only one experienced physician performed ultrasound analyses. Intraobserver variability of all ultrasound scan measurements was evaluated by means of intraclass correlation coefficient (ICC, good if >0.80) [40]. In particular, FMD measurements showed good reproducibility with an ICC of 0.95.

### 2.4. Evaluation of Carotid Intima-Media Thickness (C-IMT)

Ultrasonographic echo-color Doppler studies of left and right common carotid arteries were performed bilaterally by the same physician with a Philips Sonos 5500 using a 7.5 MHz high resolution probe. The patients were placed in supine position, with the neck extended and rotated contralaterally by 45°, and the common carotid arteries were examined on the sagittal axis with a lateral view. C-IMT was defined as a low-level echo grey band that does not project into the arterial lumen and was measured during end-diastole according to the method described by Pignoli: by focusing and freezing images on the distal wall of the common carotid artery on the lengthwise axis, the C-IMT was the distance between the leading borders of the first hyperechoic line and of the second hyperechoic line, separated by a hypoechoic space “double-track pattern” [41]. The measurements were performed bilaterally 1 cm proximally to the carotid bulb, for three times, and then C-IMT value was calculated as the arithmetical mean of each side. C-IMT measurements were always performed in arterial segment devoid of atherosclerotic plaque, defined according to Mannheim carotid intima-media thickness consensus (2004–2006) as C-IMT greater than 1.5 mm or a focal structure encroaching into the arterial lumen of at least 0.5 mm or 50% of the surrounding C-IMT value [42].

In order to reduce bias, only one experienced physician performed ultrasound analyses. ICC showed good reproducibility with a value equal to 0.98 [40].

### 2.5. Statistical Analysis

Continuous variables were expressed as means ± standard deviations and compared with ANOVA. When the single-factor ANOVA rejected the hypothesis of mean equality among the groups, Tukey test was applied for a comparison of the means of the different groups. The criterion used for this comparison was a *p* < 0.05.

Correlation between FMD and transient elastometry values was evaluated by Pearson's correlation coefficient. The correlation was considered weak for values <3, moderate for values ≥3 and <7, and strong for values ≥7. A *p* value <0.05 was considered statistically significant.

Dichotomic variables were expressed as percentages and compared with Chi-squared test. A *p* value <0.05 was considered statistically significant.

Logistic regression was performed in order to calculate odds ratio (OR); receiving operator C (ROC) curve analysis was also performed and sensitivity, specificity, and predictive power were calculated.

*Sample Size Calculation*. In the present study we planned an analysis of a continuous variable (FMD) from independent groups. Previously, we have demonstrated that, within each group of subjects, FMD is a variable that is normally distributed with a standard deviation of 1.4% [43]. Therefore, if the true difference among group means is 1.5%, we need to study groups of 15 subjects to be able to reject the null hypothesis that the population means are equal with a probability (power) of 80%. Type I error probability associated with this test of this null hypothesis is 0.05.

## 3. Results

As shown in Table 1, the three tertiles of patients were significantly different for their fibrosis degree, according to fibroscan and APRI values (*p* = 0.0001), and aspartate aminotransferase (AST) levels (*p* = 0.02). No difference was observed according to sex, age, systolic blood pressure, waist circumference, total cholesterol, triglycerides, alanine aminotransferase (ALT), HCV-RNA quantization, and prevalence of genotype 1.

**Figure 1:**
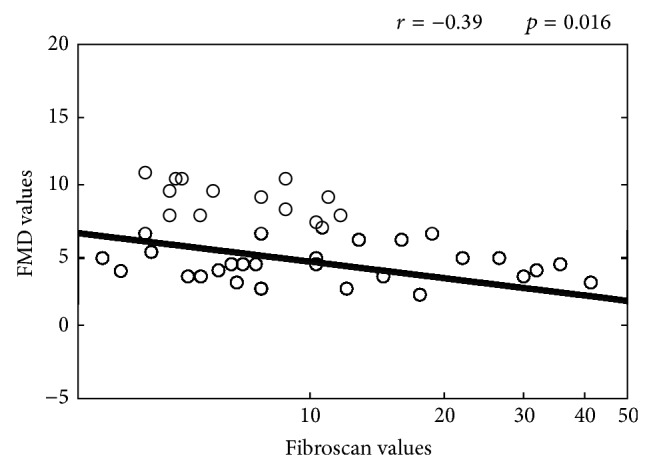
Correlation between FMD and fibroscan values.

According to FMD values, we observed a progressive statistically significant decrease from the first to the third tertile (*p* = 0.03) (Table 2). In particular, FMD in the third tertile was significantly lower as compared to the first and second tertile after performance of the Tukey test (*p* = 0.007 and *p* = 0.03, resp.). No significant difference was found between the second and first tertiles. When we evaluated the percentage of patients with endothelial dysfunction (i.e., FMD < 5%), we found a statistically significant difference among the three groups: the percentage of patients showing an FMD value <5% progressively increased with the progression of liver fibrosis (*p* = 0.04) (Table 2). Finally, Figure 1 showed a statistically significant inverse correlation between FMD and fibroscan values (*r* = −0.39, *p* = 0.016).

All C-IMT values were in the normal range (<0.9 mm) and were not statistically different among the three tertiles (0.7 ± 0.2 in I tertile, 0.7 ± 0.1 in II tertile, and 0.7 ± 0.1 in III tertile) (see Table 2).

**Figure 2:**
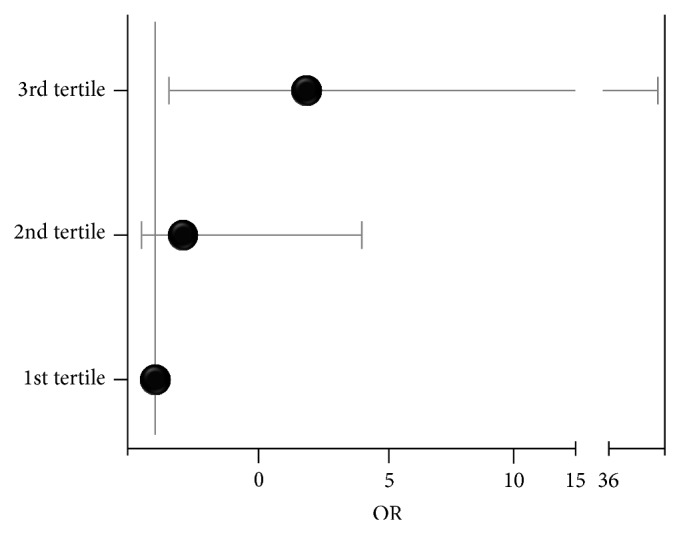
Risk of endothelial dysfunction in the three tertiles. Odds ratio (OR) = 6.9 (95% C.I. 1.4–35.1, *p* = 0.0069) comparing patients of the 3rd tertile with those of the 1st tertile.

**Figure 3:**
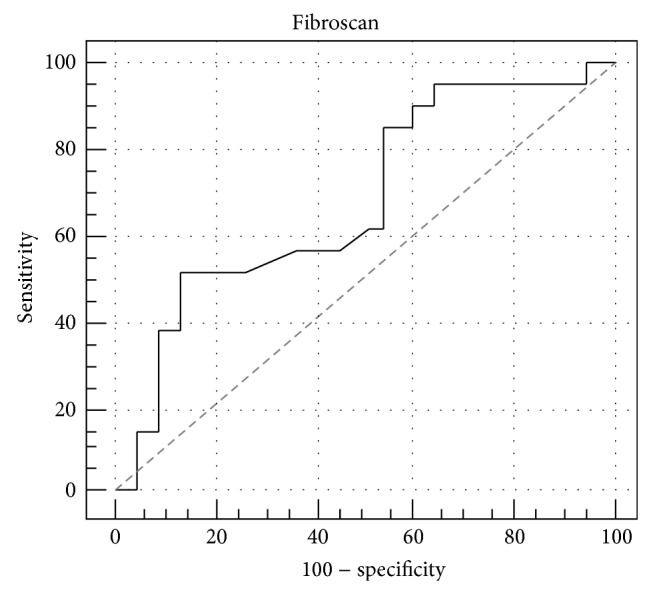
Accuracy of fibroscan in predicting endothelial dysfunction. Sensitivity 52% and specificity 86%. PPV equal to 79% and NPV of 66% in predicting endothelial dysfunction.

The data reported in Figure 2 demonstrated that the risk to develop endothelial dysfunction in patients with advanced liver fibrosis (third tertile) was 6.9 higher (95% C.I. 1.4–35.1, *p* = 0.02) than that in patients with the lowest degree of liver fibrosis (fist tertile). The odds ratio between the second and the first tertile was 1.9 but this did not reach a statistical significance (95% C.I. 0.4–9.0, *p* = 0.43). Finally, the ROC curve analysis adopted to determine the fibroscan value able to predict endothelial dysfunction found a value >11.5 KPa able to reach a positive predictive power of 79% (Figure 3).

## 4. Discussion

It has been suggested that HCV has a proatherosclerotic activity due to both its local action on the artery wall (HCV-RNA sequences have been found within the atherosclerotic plaque) [44] and stimulation of proinflammatory substances (interleukin-6 [IL-6] and tumor necrosis factor *α* [TNF*α*]) [45].

To the best of our knowledge, this is the first study aiming at evaluating endothelial dysfunction in monoinfected HCV patients (without any risk factors for CAD, with different degrees of liver fibrosis) in order to clarify the role of HCV in the initiation and progression of atherosclerotic process. In particular, the use of brachial artery FMD and C-IMT allowed us to evaluate early signs of functional and morphological alterations in vascular endothelium performance. FMD is able to offer a precious dysfunctional picture of vascular impairment as compared to C-IMT as its alterations usually develop at early stages than the morphological lesions related to carotid artery walls [46].

The exclusion criteria adopted allowed us to rule out several confounding factors from our analysis, in order to specifically evaluate the role of chronic hepatitis C in cardiovascular risk profile of apparently cardiovascular healthy individuals. The influence of demographical, serological, and anthropometric parameters was excluded since these factors were not statistically significantly different among the three groups (Table 1). The only parameter that differed in the 3 tertiles was the AST level that was significantly higher in the third tertile as compared to first and second tertile. However, such an increase, in the absence of an ALT flare (data not shown), was considered just a sign of a stable liver damage not influencing liver stiffness [47]. Moreover, fibroscan measurements correlated with APRI values in this group of patients, excluding the possibility that the former method could have overestimated the degree of fibrosis in the third tertile.

Endothelial dysfunction, expressed by FMD, was significantly influenced by the progression of liver fibrosis since average FMD values progressively decreased from the first to the third tertile. These results were further confirmed by the significant inverse correlation found between FMD and fibroscan values. A further confirmation of this datum comes from the observation that the percentage of patients with FMD < 5% significantly increased from the first to the third tertile groups.

We also calculated the risk to develop endothelial dysfunction in the third tertile that was 6.9-fold higher as compared to that of first tertile. Finally, a fibroscan value >11.5 KPa, which represents the cut-off for patients in the third tertile, was highly predictive of endothelial dysfunction.

Masiá et al. [31] already studied the relationship between FMD and HCV infection. Nevertheless, they performed their evaluations in patients suffering from HCV-HIV coinfection, at different stages of liver fibrosis, while we enrolled only mono-HCV infected patients. They found similar FMD values when comparing HCV-HIV coinfected with HIV monoinfected patients, whereas circulating adhesion molecules (CAM), considered as indirect marker of endothelial dysfunction, were increased in presence of HCV [31]. Another study exploring endothelial dysfunction, conducted in patients with HCV-HIV coinfection, at different stages of liver fibrosis, concluded that HCV-HIV coinfected patients have higher levels of CAM as compared with healthy subject and that these levels were higher in the presence of HCV genotype 1 and advanced fibrosis [32].

We excluded the presence of a clear morphological alteration in vascular walls as demonstrated by the C-IMT values in the three groups. This result is in contrast with literature data [9–12]. Nevertheless, the absence of a pathological C-IMT at the different stages of liver fibrosis in our population can be attributable to the strict selection criteria able to detect a completely healthy population according to cardiovascular risk profile. The higher prevalence of atherosclerotic damage found in patients with chronic HCV infection in other literature studies might be related to the broad inclusion criteria adopted in other researches and, therefore, to confounding factors able to negatively influence the results of previous researches [9, 11, 12].

Our findings attribute a pathogenetic role to HCV infection in the genesis of the atherosclerotic process by means of the chronic inflammation and the development of liver fibrosis related to virus infection. Nevertheless, it seemed that the inflammatory systemic process induced by HCV was not able by itself to determine a morphological arterial damage, while it was able to predispose endothelium to early impairment of its inner functions.

A limitation of our study is represented by the fact that we did not evaluate inflammatory markers (such as C-reactive protein) and the dosage of CAM in our patients. However, the goal of this study was to correlate FMD and C-IMT with the stage of liver fibrosis.

The small sample size of our population is a further limitation of our research. Nevertheless, it should be considered that we originally considered 380 patients suffering from HCV-related chronic hepatitis. The need to reduce confounding factors led us to adopt strict selection criteria that enormously reduced the number of the sample size of our population although this allowed us to have results free from biases.

## 5. Conclusions

HCV-related chronic hepatitis has a role in the atherosclerotic process since it induces endothelial dysfunction, an early sign of atherosclerotic process development in patients free from other cardiovascular risk factors.

## Figures and Tables

**Table 1:** Demographic, serological, and anthropometric parameters, systolic blood pressure, and stage of fibrosis assessed by fibroscan and APRI, in the 3 tertiles.

	I tertile	II tertile	III tertile	*p*
Number of pts.	15	15	15	
M/F	6/9	10/5	8/7	0.32^*^
Age	59.7 (11.8)	61.5 (9.4)	65.6 (8.1)	0.28
BMI	25.1 (3.7)	26.5 (2.1)	27.1 (3.5)	0.20
Waist circumference (cm)	87.1 (13.4)	90.6 (10.3)	92.8 (9.1)	0.35
Total cholesterol (mg/dL)	171.5 (23.4)	165.2 (20.7)	150.8 (31.6)	0.09
Triglycerides (mg/dL)	75.3 (23.7)	97.7 (59.8)	86.9 (47.5)	0.44
ALT^†^	33.2 (22.4)	72.4 (86.0)	68.8 (56.7)	0.17
AST^†^	36.1 (14.3)	55.5 (34.9)	69 (40.2)	0.02^‡^
HCV-RNA genotype 1	66.7%	73.3%	73.3%	0.89^*^
Quantitative HCV-RNA (UI/mL)	3,718,195 (7,587,287)	1,300,529 (2,267,567)	2,511,572 (5,442,431)	0.51
Systolic blood pressure (mmHg)	126.9 (10.5)	129.0 (10.2)	129.6 (11.4)	0.81
APRI	0.4 (0.1)	0.7 (0.4)	1.5 (1.0)	<0.001^§^
Fibroscan (Kpa)	4.9 (0.8)	8.6 (1.4)	27.2 (12.1)	<0.001^§^

The values reported in the table represent the mean (SD) or the percentages obtained in all patients.

^*^By Chi-Squared test.

^†^Normal value: 40 UI/L.

^‡^ANOVA demonstrated a significant difference among the 3 groups. I tertiles ≠ II tertiles; I tertiles ≠ III tertiles by Tukey test.

^§^ANOVA demonstrated a significant difference among the 3 groups. I tertiles ≠ II tertiles ≠ III tertiles by Tukey test.

**Table 2:** FMD value, percentage of patients with a pathological FMD value, and c-IMT in the three tertiles.

	I tertile	II tertile	III tertile	*p*
Number of pts.	15	15	15	
FMD: mean (SD)	7.1 (2.8)	6.1 (2.4)	4.7 (1.7)	0.03^*^
Pts. with FDM <5%	27%	43%	73%	0.04^†^
C-IMT (mm)	0.7 ± 0.2	0.7 ± 0.1	0.7 ± 0.1	ns

^*^ANOVA demonstrated a significant difference among the 3 groups. I tertiles ≠ III tertiles and II tertiles ≠ III tertiles by Tukey's test.

^†^Statistical analysis by Chi-squared test.
